# Institutional Outcomes Should Be a Determinant in Decision to Perform Laparoscopic Proctectomies for Rectal Cancer

**DOI:** 10.7759/cureus.7666

**Published:** 2020-04-14

**Authors:** Asya Ofshteyn, Allson B Weaver, Justin T Brady, Jay Idrees, Wendy M Coronado, Scott R Steele, Harry Reynolds, Emily Steinhagen, Sharon L Stein

**Affiliations:** 1 Surgery, University Hospitals Cleveland Medical Center / University Hospitals Research in Surgical Outcomes & Effectiveness Center (UH-RISES), Cleveland, USA; 2 General Surgery, University Hospitals Cleveland Medical Center / University Hospitals Research in Surgical Outcomes & Effectiveness Center (UH-RISES), Cleveland, USA; 3 Colorectal Surgery, Cleveland Clinic, Cleveland, USA; 4 Surgery, Harney District Hospital, Burns, USA; 5 Colorectal Surgery, University Hospitals Cleveland Medical Center / University Hospitals Research in Surgical Outcomes & Effectiveness Center (UH-RISES), Cleveland, USA

**Keywords:** laparoscopic, proctectomy, institutional outcome, rectal cancer, minimally invasive surgery

## Abstract

Purpose

Minimally invasive rectal cancer (RC) resection has become common, despite recent high-profile trials failing to show non-inferiority to open proctectomy. We hypothesized that at a high-volume center, laparoscopic resection may have superior outcomes compared to those seen in ALaCaRT and ACOSOG Z6051.

Methods

Retrospective review of patients undergoing laparoscopic proctectomy from 2007 to 2015 for RC was performed at a high-volume center. Primary outcome was successful resection defined by negative circumferential resection margin (CRM) and distal margin (DM), and complete total mesorectal excision (TME).

Results

A total of 89 patients were included. Of 33 patients with TME grading, 31 (93.9%) had complete/near complete TME, and 29 (87.9%) had a “successful resection” compared with 81.7% in ACOSOG and 82% in ALaCART trials using same criteria. CRM was ≥1 mm in 87 (97.8%) of patients. Mean DM was 3.8 cm; 97.8% of patients had negative DM.

Conclusion

High-volume centers can achieve similar high quality RC outcomes to those demonstrated in recent trials. Institutional outcomes should determine optimal surgical technique.

## Introduction

The goal of surgical resection for rectal cancer is to optimize cancer-free survival and lower the risk of both local and distant recurrence. Due to the lack of longitudinal data, much of the current surgical research has focused on early outcomes including intactness of the mesorectum, and negative circumferential and distal margins [1-9]. There is an ongoing debate regarding the optimal operative approach for management of resectable rectal cancer. It is uncertain whether minimally invasive surgery (MIS) provides equivalent short-term and/or long-term outcomes for these patients in comparison to traditional open resection.

The CLASSIC trial found that patients undergoing laparoscopic proctectomy had a higher rate of positive circumferential margins compared to open surgery [3]. However, follow-up data failed to demonstrate an oncologic difference in long-term outcomes [10]. ACOSOG Z6051 and ALaCaRT trials failed to demonstrate non-inferiority of short-term outcomes for MIS proctectomy compared to open resection [6,7]. On recently published two-year follow-up results, both studies did not demonstrate significant differences in rates of disease-free survival or recurrence between laparoscopic and open approaches, though estimates of treatment effect in ALaCaRT favored open over laparoscopic approach [11,12]. These results have amplified concerns regarding oncologic safety of MIS approaches for rectal cancer.

High hospital volume, defined as more than 25 laparoscopic proctectomies/year, has been associated with better outcomes in rectal cancer [13]. It is therefore reasonable to expect that oncologic safety following laparoscopic proctectomy may vary based on institutional experience. While the feasibility of laparoscopic approaches for surgical resection of rectal cancer has been demonstrated in the setting of randomized trials, institutional level studies evaluating the feasibility, safety and efficacy of laparoscopic proctectomies are limited.

Our aim was to compare our experience with laparoscopic proctectomies to assess outcomes in comparison with data from ACOSOG and ALaCaRT trials. We hypothesized that a high-volume minimally invasive center, where surgeons preferentially select MIS techniques, would have better outcomes following a laparoscopic proctectomy than currently reported in the literature. This would justify a preference for laparoscopic approach in carefully selected patients based on individual center outcomes.

## Materials and methods

Following Institutional Review Board (IRB) approval, a retrospective review of patients undergoing laparoscopic resection for rectal cancer at University Hospitals Cleveland Medical Center from 2007 to 2015 was performed. Patients who underwent surgery for rectal cancer were identified from the medical record using International Classification of Disease 9th Revision diagnosis code 154.1. Inclusion and exclusion criteria were adopted from the ACOSOG and ALaCaRT trials. Patients were included if they were over 18 years of age, had a primary rectal adenocarcinoma (defined as <15 cm from the anal verge by endoscopic or radiologic measurement) diagnosed by histology and were planned to undergo laparoscopic resection. Patients were excluded if they had recurrent disease, were scheduled for a palliative resection, multivisceral resection or intraoperative radiation therapy, if they had clinical stage T4 tumors, stage IV disease or unknown preoperative clinical staging information.

Patient information collected included demographics, comorbidities, use of neoadjuvant and adjuvant chemoradiotherapy, clinical and pathological staging, operative details, complete total mesorectal excision (TME), specimen grade (when available), hospital length of stay and postoperative complications. Patients underwent clinical evaluation including proctoscopy, computed tomography, ultrasound and magnetic resonance imaging as deemed clinically appropriate. The decision for laparoscopic surgery was at surgeon discretion and preference. Study data were collected and managed using a REDCap database hosted at University Hospitals Cleveland Medical Center [14].

Primary and secondary outcomes

The primary outcome was the composite endpoint of negative distal and circumferential resection margins and TME grade, when available. This was modeled on the ALaCaRT and ACOSOG primary study endpoints. Secondary endpoints included conversion rate and significant intraoperative and postoperative morbidity (Clavien-Dindo class 3-5 complications). This data was then compared to published outcomes for rectal cancer to determine if they matched or exceeded acceptable standards for minimally invasive surgery.

Statistical analysis

Continuous variables are shown as the mean with standard deviation (SD) or median with interquartile range (IQR) and were compared using two-way Student t-test or Wilcoxon rank sum test where appropriate. Categorical variables are displayed as frequencies with percentages and were compared using Chi-square or Fischer’s exact where appropriate. A p-value less than 0.05 was considered significant. Statistical analysis was performed using Stata/SE 14.2 (StataCorp, College Station, TX, USA).

## Results

There were 386 patients who underwent minimally invasive proctectomy between 2007 and 2015 at our institution. In 148 (38.3%) cases, the proctectomy was performed for malignancy. Of these, 89 (60%) patients met the inclusion criteria and were included in the analysis. Patients with multi-visceral resection (n = 19), recurrent disease (n = 20), stage 4 disease (n = 14) and intraoperative radiation (n = 6) were excluded. Mean age at the time of surgery was 66 years and 54 (60.7%) patients were male. Nearly 80% of patients received neoadjuvant chemotherapy and radiation. Table 1 summarizes patient characteristics.

**Table 1:** Demographics and patient characteristics, current study compared to ACOSOG. SD: Standard deviation; BMI: Body mass index ^a^ Rectal tumor location was defined as follows: low – ≤5 cm from the anal verge, middle – from >5 to 10 cm, and high from >10 to 15 cm.

	Current Study	ACOSOG Z6051	P-value
Patients, n	89	242	
Male gender, n (%)	54 (60.7)	156 (64.5)	0.84
Age, mean (SD)	66.3 (12.9)	57.1 (11.5)	<0.0001
BMI, mean (SD)	28.4 (7.2)	26.4 (4.0)	0.002
Planned Operation, n (%)			0.25
Abdominal perineal resection	17 (19.1)	55 (22.7)	
Low anterior resection	71 (79.8)	187 (77.3)	
Total proctocolectomy	1 (1.1)		
Location of tumor in rectum, n (%)^a^			<0.001
High	13 (15.9)	33 (13.6)	
Middle	49 (59.8)	85 (35.1)	
Low	20 (24.4)	124 (51.2)	
Tumor distance from anal verge, mean (SD), cm	6.8 (2.9)	6.1 (3.1)	0.07
Preoperative clinical stage, n (%)			<0.001
I	17 (19.1)	2 (0.8)	
II	32 (36)	99 (40.9)	
III	40 (44.9)	141 (58.2)	
Preoperative therapy received, n (%)			<0.001
Chemotherapy + radiation	71 (79.8)	227 (95.0)	
Radiation alone	2 (2.2)	8 (3.3)	
Chemotherapy	1 (1.1)	4 (1.7)	
Unknown	0	3	
None	15 (17.6)	0	

Operative technique

Surgery was performed laparoscopically in 79 (88.8%) of the cases. Of the remaining 10 (11%) included cases, eight were robotic-assisted and two cases were hand-assisted laparoscopic resection. Included surgical procedures were low anterior resection (LAR) with anastomosis (n = 60, 67%), abdominoperineal resection (APR) (n = 23, 26%), Hartmann procedure (n = 5, 6%) and total proctocolectomy (n = 1, 1%). A stoma was created in 87 cases, 30.3% of which were colostomies. The mean operative time was 326.4 minutes, with a mean estimated blood loss (EBL) of 137.2 mL. Conversion rate was 24.7% (n = 22/89). Cases that required conversion were still analyzed as their intended MIS approach. Intraoperative complications occurred in 3.4% of surgeries.

Short-term complications within 30 days of surgery occurred in 31.5% (n = 28), most commonly including deep space infection (n = 7, 7.9%), ileus (n = 7, 7.9%), urinary retention (n = 7, 7.9%), surgical site infection (n = 6, 6.7%) and atrial fibrillation (n = 4, 4.5%). The rate of re-operation was 4.5% (n = 4) and the overall 30-day mortality was 1.1% (n = 1/89). Readmission occurred in 12.4%. The clinical outcomes are summarized in Table 2.

**Table 2:** Operative details and outcomes, current study compared to ACOSOG. EBL: Estimated blood loss.

	Current Study	ACOSOG	P-Value
Surgical Approach, n (%)			<0.001
Low Anterior Resection	3 (3.4)	68 (35.6)	
Low Anterior Resection + Coloanal Anastomosis	57 (64.0)	109 (57.1)	
Abdominal Perineal Resection	23 (25.8)	11 (5.8)	
Low Hartmann	5 (5.6)	1 (0.4)	
Total Proctectomy	0	2 (1.0)	
Total Proctocolectomy	1 (1.1)	0	
Surgical Approach			<0.001
Laparoscopic	79 (88.8)	165 (68.8)	
Hand assisted	2 (2.3)	41 (17.1)	
Robotic assisted	8 (9.1)	34 (14.2)	
Ostomy Created			0.73
Colostomy	27 (30.3)	63 (26.3)	
Ileostomy	60 (67.4)	171 (71.3)	
Sphincter preservation planned before operation, n (%)	68 (68.7)	191 (79.6)	0.62
Margins examined by frozen, n (%)	10 (11.2)	51 (21.3)	0.04
Open to close operative time, mean (SD), min	326.4 (100.2)	266.2 (101.9)	<0.0001
Total EBL, mean (SD), mL	137.2 (139.8)	256.1 (305.8)	0.0005
Conversion, n (%)	22 (24.7)	27 (11.3)	0.001
Complications (intraoperative and postoperative), n (%)	33 (37.1)	137 (57.1)	0.001
Total intraoperative complications, n (%)	3 (3.4)	26 (10.8)	0.047
Rectum	1 (1.1)	10 (4.2)	
Colon	0	3 (1.3)	
Small bowel	0	0	
Ureter	0	1 (0.4)	
Bladder	0	1 (0.4)	
Spleen	0	0	
Hemorrhage/bleeding associated with surgery	0	8 (3.3)	
Other	2 (2.2)	5 (2.1)	
Maximum grade of postoperative complications, Clavien-Dindo, n (%)	28 (31.5)	129 (53.8)	<0.001
3	7 (7.9)	46 (19.2)	0.06
4	0	6 (2.5)	
5	3 (3.4)	2 (0.8)	
Anastomotic leak during postoperative period, n (%)	0	5 (2.1)	0.17
30-day mortality, n (%)	3 (3.4)	2 (0.8)	0.09
Rehospitalization (within 30 days of discharge), n (%)	11 (12.4)	8 (3.3)	0.002
Reoperation, n (%)	7 (7.9)	12 (5.0)	0.32

Technical and oncologic success

A clear circumferential margin was obtained in 87 (97.8%) cases. A negative distal margin was achieved in 87 (97.8%) cases. Completeness of TME based on pathologic assessment was not standardized until 2012; therefore, TME completeness was only available for 33 cases. The 33-patient cohort was statistically compared to our larger 89-patient group and was found to be clinically similar in the relevant demographic, patient and tumor characteristic, operative detail and outcome parameters. TME was graded as complete/near complete in 31 (93.9%) of the cases (29 complete, four nearly complete). The mean number of lymph nodes examined was 18.3 (SD 5). Overall, 87.9% of cases were considered “pathologically successful” based on negative circumferential and distal margins and complete/near complete TME. The majority of tumors (69.7%) were well or moderately differentiated. These results are summarized in Table 3.

**Table 3:** Oncologic outcomes, current study compared to ACOSOG. CRM: Circumferential resection margin; TME: Total mesorectal excision. ^a ^Circumferential resection margin for present study defined as negative if >1 mm, for ACOSOG study as >1 mm. ^b ^For 33 patients in current study with TME grade listed. Defined as complete TME, negative circumferential and distal margins.

	Current Study	ACOSOG Z6051	P-value
Circumferential Resection Margin, n (%)^a^			<0.001
Negative CRM	87 (97.8)	211 (87.9)	
Positive	1	29	
Distance to nearest radial margin, mean (SD), mm	11 (8.4)	12.8 (11.2)	0.28
Negative distal margin, n (%)	87 (97.8)	236 (98.3)	1.0
Distance to distal margin, mean (SD), cm	3.8 (3.7)	3.2 (2.6)	0.1
Total Mesorectal Excision, n (%)	(n = 33)	(n = 240)	0.14
Complete	29 (87.9%)	175 (72.9)	
Nearly complete	4 (12.1%)	46 (19.2)	
Incomplete	0	19 (7.9)	
Number of lymph nodes examined, mean (SD)	18.3 (5.0)	17.9 (10.1)	0.72
Successful resection^b^	87.9%	81.7%	0.47
Total length of resected specimen, mean (SD), cm	27.7 (12.6)	28.9 (10.8)	0.36
Stage, n (%)		239	0.16
0	13 (14.6)	55 (23)	
I	40 (44.9)	76 (31.8)	
II	18 (20.2)	47 (19.6)	
III	18 (20.2)	60 (33.5)	
IV	0	1 (0.4)	
Tumor size, mean (SD), cm	2.1 (1.5)	2.3 (1.8)	0.37

Outcomes compared to published literature

We compared our data to historical data provided from the ACOSOG and ALaCaRT trials. Demographic data between our study and the ACOSOG and ALaCaRT studies was generally similar, with a few noticeable differences (Table 4). In our experience, the location of the tumor was slightly higher in the rectum, with only 24.4% being low compared to 51.2% for the ACOSOG and 35% for the ALaCaRT. We also had fewer patients who received preoperative chemoradiation (79.8%) compared to the 95% of ACOSOG, but more than the 50% of ALaCaRT participants who received preoperative radiation.

**Table 4:** Demographics and patient characteristics, current study compared to ALaCaRT. IQR: Interquartile range.

	Current Study	ALaCaRT	P-value
Male gender, n (%)	54 (60.7)	160 (67)	0.3
Age, median (IQR), y	68 (57-750)	65 (56-74)	
BMI, median (IQR)	28.4 (24.1-33.7)	27 (24-30)	
BMI > 30, n (%)	45 (60)	56 (24)	<0.0001
Location of tumor in rectum, n (%)			0.04
High	13 (15.9)	53 (22)	
Middle	49 (59.8)	103 (43)	
Low	20 (24.4)	82 (35)	
Tumor stage, n (%)			0.13
T1	1 (1.0)	18 (8)	
T2	21 (23.6)	68 (29)	
T3	67 (75.3)	151 (63)	
Nodal status, n (%)			0.001
N0	48 (53.9)	107 (45)	
N1	39 (43.8)	92 (39)	
N2	2 (2.3)	37 (16)	
Distant metastases, n (%)	0	10 (4)	0.007
Preoperative radiotherapy, n (%)	73 (82.0)	119 (50)	<0.001

Operative data varied between our study and ACOSOG. While our operative time was significantly longer (326.4 min vs. 266.2 min, p < 0.0001), other intraoperative parameters were favorable including lower EBL (137.2 vs. 256.1, p = .0005), fewer intraoperative complications (37.1% vs. 57.1%, p = .047), and lower rate of postoperative complications (31.5% vs. 53.8%, p < .001). Clinically, a higher proportion of our patients underwent APR (25.8% vs. 5.8% p < 0.001) and our study had a lower proportion of hand-assisted or robotic assisted procedures (11.4% vs. 31.3% p < 0.001) than the ACOSOG trial. Compared to ACOSOG, conversion rate (24.7% vs. 11%, p = 0.001) and 30-day readmission rates (12.4% vs. 3.3%, p = 0.002) were higher in our experience (Table 5).

**Table 5:** Operative details and outcomes, current study compared to AlaCaRT. IQR: Interquartile range; EBL: Estimated blood loss.

	Current Study	ALaCaRT	P-value
Surgical Approach, n (%)			<0.001
Low Anterior Resection	3 (3.4)	143 (60)	
Low Anterior Resection + coloanal anastomosis	57 (64.0)	69 (29)	
Abdominal Perineal Resection	23 (25.8)	25 (11)	
Low Hartmann	5 (5.6)	0	
Total Proctectomy	0	0	
Total proctocolectomy	1 (1.1)	0	
Ostomy Created, n (%)			0.003
Colostomy	27 (30.3)	30 (13)	
Ileostomy	60 (67.4)	162 (68)	
Operative time, median (IQR), min	310 (253-380)	210 (163-253)	
Total EBL, median (IQR), mL	100 (22.5-200)	100 (50-200)	
Conversion, n (%)	22 (24.7)	21 (9)	<0.001

Operative data for our study was similar to ALaCaRT data. A lower proportion of our patients underwent LAR (67.4% vs. 89%, p < 0.001), and our operative times were comparatively longer (310 vs. 210 minutes). EBL was similar between the studies. The rate of conversion to open surgery was statistically higher in our study (24.7% vs. 9%, p < 0.001). Other outcome measures in ALaCART were reported as medians and thus comparisons could not be performed (Table 6).

**Table 6:** Oncologic outcomes, current study compared to ALaCaRT. IQR: Interquartile range

	Current Study	ALaCaRT	P-value
Radial margin, n (%)			0.049
Positive (<1 mm)	1 (1.1)	16 (6.7)	
Negative (> or equal to 1 mm)	87 (98.9)	222 (93)	
Negative distal margin >1 mm, n (%)	87 (97.8)	236 (99)	0.3
Total Mesorectal Excision, n (%)			0.66
Complete	29 (87.9)	206 (87)	
Nearly complete	4 (12.1)	24 (10)	
Incomplete	0	8 (3)	
Successful resection, n (%)	29 (87.9)	194 (82)	0.47
Tumor stage, n (%)			0.22
T0 (or no residual tumor)	12 (13.5)	33 (14)	
T1	13 (14.6)	23 (10)	
T2	31 (34.8)	67 (28)	
T3	31 (34.8)	104 (44)	
T4	1 (1.1)	11 (5)	
Tumor size, median (IQR), mm	20 (10-30)	30 (20-40)	
Total length of resected specimen, median (IQR), mm	260 (215-313)	260 (205-310)	

Overall, our pathologic outcomes were favorable when compared to ACOSOG results. Our CRM margin was negative in 97.8% compared to the 87.9% of ACOSOG (p < .001). The overall “success rate” was higher in our study compared to in ACOSOG (87.9% vs. 81.7%, p = 0.47), although this value did not reach statistical significance. Lymph node harvests were similar between studies.

Pathologic outcomes between our study and ALaCaRT were similar for most measures, including negative distal margin, completeness of TME, and percent successful resection. Our radial margin was negative in 98.9% of patients, compared to 93% (p = 0.047) in the ALaCaRT data. The tumors in the two studies had similar sizes and histologic grades, although a greater proportion of patients in our study had node negative disease (N0 79.8% vs. 62%, p < 0.01).

## Discussion

This study demonstrates that at a high-volume minimally invasive center, MIS proctectomy for surgical resection of rectal cancer can be performed safely with excellent short-term oncologic outcomes. While much of the current debate regarding the use of MIS for resection of rectal cancer is focused on the feasibility of technique, we believe that the preference for minimally invasive approaches should be based on institutional and surgeon data.

To this point, we found that the selection of a minimally invasive approach was justified in our experience as we were able to achieve a high overall negative CRM margin in 98% of the cases and an overall success rate of 88% in the 33-patient cohort with reported TME grade. While this success rate surpasses 81.7% in ACOSOG and 82% in ALaCART, it is comparable to the open surgical success seen in ACOSOG (86.9%) and ALaCART (89%). We have demonstrated that individual centers can achieve results that compare favorably to the outcomes of MIS approaches and are similar to results of open approaches reported in ACOSOG and ALaCaRT clinical trials. These results emphasize the importance of institutional level evaluation of outcomes to guide decision making regarding the appropriate surgical approach. We believe that our good outcomes can be attributable to careful patient selection of best candidates for surgery and high volume of experience of each surgeon practicing in our institution at the time.

The findings from our study are consistent with the current literature and adds to the growing evidence in favor of the use of laparoscopic approaches. Arezzo et al. performed a systematic review and meta-analysis, evaluating the outcomes of laparoscopic approaches compared to open resection [9]. In their meta-analysis they included results from clinical trials and retrospective studies and found that the incidence of CRM margin involvement was 7.9% after laparoscopic resection compared to 6.9% in the open resection group with an overall relative risk of 1.0 (95% CI: 0.73-1.35). The authors concluded that the oncological adequacy of laparoscopic proctectomy was equivalent to open resection. Another, more recent meta-analysis by Acuna et al. demonstrated non-inferiority of laparoscopic surgery compared to open in terms of quality of surgical resection outcomes, including positive CRM, incomplete TME and positive distal resection margin [15].

In our experience, the CRM margin involvement was even lower than demonstrated by Arezzo et al. (2.2%), indicating that there can be significant variability in outcomes and practice patterns among institutions. While many studies, such as ACOSOG and ALaCaRT, compare data to equivalent patients who undergo open surgery, we were unable to perform such comparison. At our institution, surgeons have historically preferred minimally invasive techniques for rectal cancer. In general, the open approach was reserved for more advanced tumors, hostile abdomens, or patients with severe medical comorbidities complicating minimally invasive techniques. Patients who are not selected for minimally invasive surgery are therefore not equivalent, with increased concerns for threatened margin, advanced tumors, or other contraindications to minimally invasive surgery.

There are several limitations to this study. Standardization of surgeon and pathologist oncologic evaluation over time presents complications in normalizing the data. We were only able to report completeness of TME in under half of our procedures. Therefore, we were only able to report “successful resection” in a proportion of our patients. It may be inaccurate to extrapolate high rates of complete TME to our earlier patients. The lack of uniformity in oncologic outcomes between ACOSOG and ALaCaRT complicates direct comparison. Examples of this include the use of means versus medians, and distal resection margin of 1 mm versus greater than 1 mm. Additionally, our study cohort is limited by its sample size, which restricts our ability to demonstrate statistical significance in some cases. Lastly, there were differences in tumor demographics between our study and ACOSOG and ALaCaRT. A greater proportion of our tumors were higher in the rectum, which may affect rates of “successful” surgery. Additionally, our conversion and APR rates were higher than the trial populations. Surgeon specific and institutional specific data should not be extrapolated to other institutions, and surgeons need to know their own data to make decisions about the best possible approach in each surgeon’s hands.

Decisions regarding choice of surgical technique should be based on data obtained from the institution and surgeon, rather than based on technique. Similar to baseball, one would not give a bat a batting average, the batting average would be attributed to the batter, using their bat of choice. Comparably, we hope that the debate regarding the use of minimally invasive surgery will focus on surgeon and institutional outcomes, rather than global evaluation of the techniques.

## Conclusions

Minimally invasive proctectomy for the surgical resection of rectal cancer can be performed safely, but the decision for the selection of minimally invasive approach should be based on the institutional evaluation of outcomes for optimal results.
